# Nutritional Intervention as a Complementary Neuroprotective Approach against Propionic Acid-Induced Neurotoxicity and Associated Biochemical Autistic Features in Rat Pups

**DOI:** 10.3390/metabo13060738

**Published:** 2023-06-09

**Authors:** Sana Razhan M. Alsubaiei, Hanan A. Alfawaz, Ramesa Shafi Bhat, Afaf El-Ansary

**Affiliations:** 1 Department of Food Science and Nutrition, College of Food & Agriculture Sciences, King Saud University, Riyadh 11495, Saudi Arabia; 435204014@student.ksu.edu.sa (S.R.M.A.); halfawaz@ksu.edu.sa (H.A.A.); 2 Biochemistry Department, Science College, King Saud University, Riyadh 11495, Saudi Arabia; rbhat@ksu.edu.sa; 3 Central Research Laboratory, Female Campus, King Saud University, Riyadh 11495, Saudi Arabia

**Keywords:** autism spectrum disorder, propionic acid, probiotics, *Lacticaseibacillus rhamnosus*, artichokes, luteolin, antioxidants, anti-inflammatory

## Abstract

Since there is no known cure for autism spectrum disorder (ASD), its incidence rate is on the rise. Common comorbidities like gastrointestinal problems are observed as common signs of ASD and play a major role in controlling social and behavioral symptoms. Although there is a lot of interest in dietary treatments, no harmony exists with regard to the ideal nutritional therapy. To better direct prevention and intervention measures for ASD, the identification of risk and protective factors is required. Through the use of a rat model, our study aims to assess the possible danger of exposure to neurotoxic doses of propionic acid (PPA) and the nutritional protective effects of prebiotics and probiotics. Here, we conducted a biochemical assessment of the effects of dietary supplement therapy in the PPA model of autism. We used 36 male Sprague Dawley albino rat pups divided into six groups. Standard food and drink were given to the control group. The PPA-induced ASD model was the second group; it was fed a conventional diet for 27 days before receiving 250 mg/kg of PPA orally for three days. The four other groups were given 3 mL/kg of yoghurt daily, 400 mg/Kg of artichokes daily, 50 mg/kg of luteolin daily and *Lacticaseibacillus rhamnosus* GG at 0.2 mL daily for 27 days before being given PPA (250 mg/kg BW) for three days along with their normal diet. All groups had their brain homogenates tested for biochemical markers, which included gamma-aminobutyric acid (GABA), glutathione peroxidase 1 (GPX1), glutathione (GSH), interleukin 6 (IL-6), interleukin 10 (IL-10) and tumor necrosis factor-alpha (TNF). When compared with the control group, the PPA-induced model presented increased oxidative stress and neuroinflammation but groups treated with all four dietary therapies presented improvements in biochemical characteristics for oxidative stress and neuroinflammation. As all of the therapies show sufficient anti-inflammatory and antioxidant effects, they can be used as a useful dietary component to help prevent ASD.

## 1. Introduction

Consumers’ health and wellbeing have received a lot of recent attention. As a result, high-value food products are being produced and utilized as biofunctional foods more frequently [1]. In order to prevent numerous diseases while maintaining consumer acceptance, dairy food companies, in particular, have been researching new products as functional foods with enhanced biofunctional activities.

Autism spectrum disorder (ASD) is a well-known neurodevelopmental illness with significant medical variance. It has become increasingly clear that neuroinflammation plays a part in ASD, and earlier research has shown that patients with ASD have neuroinflammation in their cerebral cortex, cerebellum, and white matter. Additionally, live patients with ASD had considerably greater proinflammatory cytokine profiles in their serum and cerebrospinal fluid (CSF) [2,3]. Numerous brain functions are significantly impacted by two-way communication between the brain and gut flora, so-called the gut-brain axis. These procedures include oxidative stress, neuroinflammation, glutamate excitotoxicity, blood–brain barrier development, neurogenesis, maturation of the microglia, synthesis of GABA, noradrenaline (NA), and dopamine (DA), and variance in behaviors, such as social interaction, which is the central aspect of ASD [4,5,6]. Autism has no known cure, thus treatments often concentrate on speech and behavioral interventions to correct the disorder’s distinctive social, behavioral, and communication deficits [7]. Common comorbidities including gastrointestinal (GI) disturbances are thought to contribute to the etiology of ASD as well as being one of its symptoms [8]. Growing evidence suggests that ASD patients have changed gut microbiota, with different alterations observed at different taxonomic levels, underscoring the significance of taking the gut-brain axis into account when treating these conditions [9]. Numerous studies using autistic people and animals have established the existence of gut microbial dysbiosis. These studies showed that aberrant bacterial species favor the gut environment in ASD. It has been demonstrated that biopsy samples from kids with ASD include aberrant Firmicutes-to-Bacteroidetes ratios [9]. Compositional dysbiosis was associated with the imbalance between these two bacterial families that differed among the gut’s distinct compartments [10]. Patients with ASD constantly exhibit an imbalance or increase in clostridium, proteobacteria, lactobacillus, desulfovibrio and Bacteroides levels [11]. Patients with ASD frequently have an unbalanced dysfunction. This is typically accompanied by decreased abundances of bifid bacterium, dialister, prevotella, veillonella, and Turicibacter [12]. In order to treat GI and behavioral symptoms, the majority of ASD patients use dietary therapies, both with and without clinical management. Although there is a lot of interest in dietary interventions, the best nutritional intervention options have not been determined [13].

Dietary habits and food preferences are also believed to play a significant role in the emergence of ASD. The significance of dietary composition in regulating or lowering ASD symptoms has been addressed in recent reviews. It is common knowledge that eating prebiotics and probiotics provides a number of health benefits by altering the gut microbiota in a good way. Autistic patients have disturbed gut microbiota which can be regulated by taking probiotics, prebiotics, and synbiotics in order to manage symptoms. [14,15]. The severity of social interactions in those with ASD shows a promising improvement in correlation with the growth of beneficial bacteria to compete with the pathogenic flora in intestines and improve recurrent GI complications, despite the rarity of studies examining the supplementation of probiotics and prebiotics in people with ASD [13,15]. This suggests that both pre- and probiotics are promising complementary medicines. The use of inulin as a prebiotic for the selective growth of the good gut bacteria lactobacilli and bifidobacteria, which are connected to a number of health advantages, is supported by growing scientific data [16]. According to Costabile et al. [17] the composition of the human fecal microbiota was significantly influenced by the daily consumption of inulin derived from globe artichokes. All artichoke genotypes have great nutritional value and are strongly advised as a component of a healthy and balanced diet, despite the fact that there was a noticeable diversity in chemical makeup and nutritional value among them [18]. It is well known that lutein (3′,4′,5,7-tetrahydroxyflavone) is a prevalent substance in plants. Plants high in luteolin have been used ethnopharmacologically to relieve inflammation. Studies employing various models have shown that luteolin supplements and extracts from luteolin-rich plants, such as artichokes, have anti-inflammatory properties [19]. Yogurt, fermented milk, and fermented vegetables are all widely acknowledged to be excellent sources of probiotics [20]. Consuming probiotics may help with the improvement of neurological and neurodevelopmental problems like ASD because it has been demonstrated that the gut microbiota and the brain share a bidirectional communication route [21]. The development of ASD characteristics is thought to be influenced by the generation of short-chain fatty acids (SCFAs) by gut clostridia and desulfovibrio, such as PPA [22]. Exposure to PPA, such as subcutaneous, intragastric gavage, intraperitoneal, and intracerebroventricular ones, have caused autistic-like symptoms in mouse models of ASD, including behavioral and physiological abnormalities, supporting the idea that PPA plays a role in the etiology of ASD [23,24,25]. 

A doctor may recommend dietary changes that focus largely on the gut microbiome since, as previously stated, research suggests that the bacterial makeup of the gut may be linked to both GI and neurological ASD symptoms [12,13]. The link between autism and food also opens up the possibility of early dietary intervention that could protect against the development of autism or prevent autism symptoms. This idea opens up exciting new avenues for research into dietary supplement therapy, which may improve the quality of life for people with autism and their families. This information sparked our interest in researching how luteolin and lactobacillus, either as supplements or in food-rich sources like artichokes or yogurt, can reduce specific biochemical variables related to oxidative stress, neurochemistry, and neuroinflammation, which are three major etiological mechanisms of ASD. We also wanted to investigate biomarkers of PPA-induced neurotoxicity in rodent models of ASD. It is important to stress that the gut microbiomes of ASD animal models that had been caused by PPA were markedly improved by the same nutritional therapies (unpublished work under review). Based on this fact, the identification of risk and protective factors is necessary to effectively target ASD prevention and intervention efforts. Our work uses a rat model to evaluate the potential risk of exposure to neurotoxic levels of PPA as well as the nutrient-protective benefits of prebiotics (artichoke & Luteolin) and probiotics (yogurt & *Lacticaseibacillus rhamnosus* GG).

## 2. Materials and Methods

In March 2019, fresh *Cynara scolymus L.* (artichoke) was purchased from local stores in Riyadh, Saudi Arabia (SA), imported from the Netherlands. Yogurt was collected for from a general store in Riyadh city. All the samples were stored at 4 °C according to Wauquier et al. [26]. Swanson Health Products, Fargo, ND, USA, provided probiotic *L. rhamnosus GG* and prebiotic luteolin supplements.

### 2.1. Extract from Cynara Scolymus L. (Artichoke) 

The heads of Cynara scolymus L. were split into petal, choke, and heart portions. Samples were washed, chopped, dried at room temperature, and then ground to a fine powder. Powder from different parts were separated individually over 72 h using an orbital shaker at 150 rpm and methanol/water (80/20, *v*/*v*). It was then extracted four more times using a new solvent (methanol/water) after being filtered using Whatman paper. The flask containing the artichoke extract was submerged in water for the duration of the evaporation procedure. A few drops of chloroform were added to ensure that all of the methanol had completely evaporated after 24 h in the hood. Until use, the final dry extract was stored at 4 °C [27].

### 2.2. Animals

Thirty-six male Sprague Dawley albino rat pups, who were around three weeks old and weighed an average of 70 g and 20 g, were used. They were randomly allocated into six groups. For the duration of the experiment, the control group was provided a conventional diet and only water. The second group was the propionic acid (PPA)-induced ASD model which were fed on standard diet for 27 days and then received 250 mg/kg of PPA orally for three days. The four other groups were fed a conventional diet for 27 days while also receiving oral administration of 3 mL/kg of yoghurt daily, 400 mg/Kg of artichokes daily, 50 mg/kg of luteolin daily, and *Lacticaseibacillus rhamnosus* GG at 0.2 mL daily for 27 days followed by the oral administration of PPA (250 mg/kg BW) for three days (days 28–30) [28,29]. All groups had their brain homogenates tested for biochemical markers GABA, GSH, GPX1, TNF-α, IL6, and IL10. 

The protocol of this study was approved by the Ethical Committee of Bioethics at King Saud University (KSU), Ref Number: SE-19-142. The experimental work was carried out in compliance with the ARRIVE guidelines. 

### 2.3. Brain Tissue Homogenates

All animals were deeply anesthetized at the end of feeding trial and killed to collect the brain tissue. The brain tissues were homogenized in bi-distilled water (1:10, *w*/*v*), and stored at −20 °C until biochemical analyses.

### 2.4. Biochemical Analyses

#### 2.4.1. Assay of Brain GSH

GSH was measured by a competitive ELISA kit (GPX1; Cat. No. CEA294Ge; Cloud Clone Corp., Houston, TX, USA), 23603 W. Fernhurst Dr., Unit 2201, Katy, TX, USA. In accordance with the manufacturer’s protocols, the measurement was completed. Less than 0.52 ug/mL were the sensitivity values.

#### 2.4.2. Assay of GPX1

GPX1 was measured by a competitive ELISA kit (GPX1; Cat. No: SEA295Ra; Cloud Clone Corp.), 23603 W. Fernhurst Dr., Unit 2201, Katy, TX, USA. In accordance with the manufacturer’s protocols, the measurement was completed. Less than 0.61 ng/mL was the sensitivity level.

#### 2.4.3. Assay of GABA

GABA was measured by a competitive ELISA kit (GPX1; Cat. No. CEA900Ge; Cloud Clone Corp.), 23603 W. Fernhurst Dr., Unit 2201, Katy, TX, USA. The method was carried out in accordance with the manufacturer’s protocols. Less than 2.17 pg/mL was the sensitivity level.

#### 2.4.4. Assay of IL-6

IL6 was measured by a competitive ELISA kit (GPX1; Cat. No: SEA079Ra; Cloud Clone Corp.), 23603 W. Fernhurst Dr., Unit 2201, Katy, TX, USA. The measurement was performed according to the manufacturer’s protocols. The sensitivity level was less than 3.3 pg/mL.

#### 2.4.5. Assay of IL-10

IL-10 was measured by a competitive ELISA kit (GPX1; Cat. No. SEA056Ra; Cloud Clone Corp.), 23603 W. Fernhurst Dr., Unit 2201; Katy; TX; USA. The test was conducted in accordance with the manufacturer’s instructions. Less than 5.8 pg/mL was the sensitivity level.

### 2.5. Statistical Analyses

The study’s findings were presented as means of variables ± standard deviations. Using SPSS version 16.0, all statistical comparisons between the control group and the PA- and nutritionally protected groups of rats were carried out using a one-way analysis of variance (ANOVA) tests together with Dunnett’s test for multiple comparisons. The threshold for significance was set at *p* < 0.05. Analysis of the ROC curve was also carried out. Calculations were made to determine the AUCs, levels of sensitivity and specificity, and cutoff values. An AUC of 0.5 often indicates no judgment (i.e., the capacity of the measured variable to confirm the neurotoxic effect of PPA and/or the protective effects of the nutritional intervention), 0.7 to 0.8 is seen as acceptable, 0.8 to 0.9 is regarded as excellent, and more than 0.9 is regarded as remarkable.

## 3. Results

**Figure 1:**
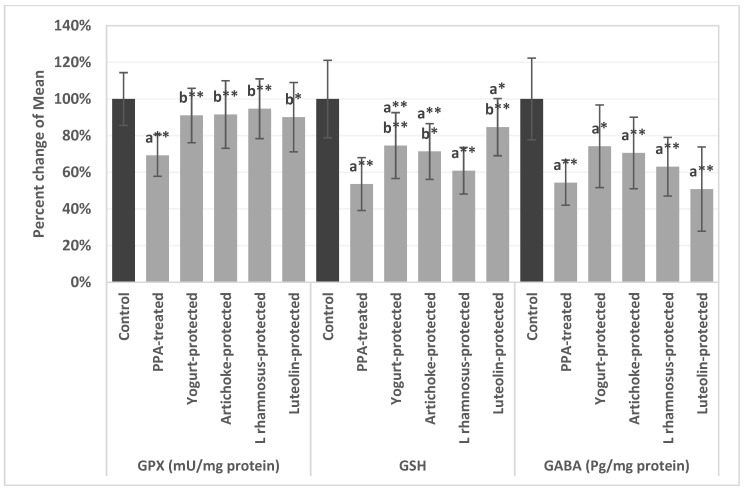
The percentage change ± error bars in GPX, GSH, and GABA levels in brain homogenates of an untreated PPA-induced autism model and nutritionally treated groups of yoghurt, artichoke, *L. rhamnosus*, and luteolin relative to control, presented as 100%. ^a^ describes significant difference to the (Control group). ^b^ describes significant difference to the (PPA-treated group). * describes significant difference at significance level (0.05). ** describes significant difference at significance level (0.01).

Table 1 presented the effect of yogurt, artichokes, luteolin, and *L. rhamnosus* on the levels of GPX1, GSH, and GABA in brain homogenates of PPA-induced animal models of autism. Figure 1 shows the percentage changes of the variables presented in Table 1. The significant decrease (*p* < 0.05) in GPX (30.76%), GSH (46.44%), and GABA (45.68%) in the PPA-induced autism rat model could be interpreted as a neurotoxic effect of PPA. Our results show the remarkable benefits of the four nutritional therapies presenting only 9, 8.4, 5.3, and 9.9% lower activity in GPX yogurt, artichoke, *L rhamnosus* GG, and luteolin, respectively, compared to control). All four nutritional interventions showed an increased level of GPX but *L rhamnosus* GG was the most effective, followed by artichokes, yogurt and luteolin, when compared to the PPA-induced ASD model (Table 1). *L rhamnosus* GG- was less effective at restoring GSH levels in PPA-treated animals, recording a value of 9.24 ± 1.35, which is still significantly lower (*p* < 0.05) than the control group (15.18 ± 2.27), while the luteolin-treated group did not show any improvement in GABA levels, recording a value of 33.51 ± 14.19 against the PPA treatment (35.80 ± 5.82).

**Figure 2:**
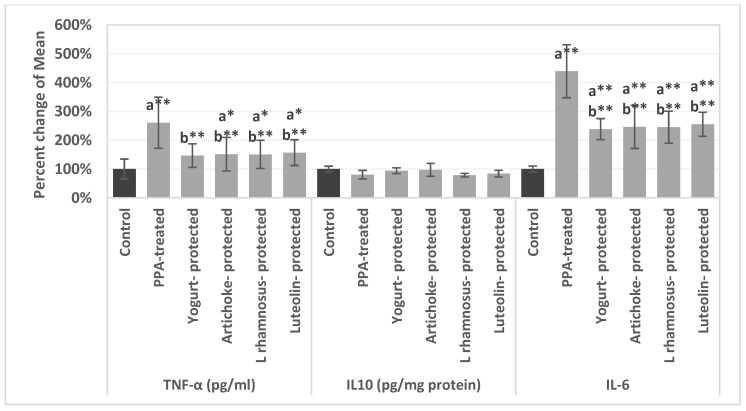
Percentage change ± error bars of TNF-α, IL-10, and IL-6 levels in brain homogenates of an untreated PPA-induced autism model and nutritionally treated groups of yogurt, artichoke, *L. rhamnosus*, and luteolin relative to control, presented as 100%. ^a^ describes significant difference with the (Control group). ^b^ describes significant difference with the (PPA-treated group). * describes significant difference at significance level (0.05). ** describes significant difference at significance level (0.01).

Table 2 shows the protective effects of nutrition with yogurt, artichoke, *L. rhamnosus*, and luteolin on the levels of TNF-α, IL10, and IL-6 in brain homogenates of the PPA-induced rodent model of autism. Figure 2 shows the percentage changes of the variables presented in Table 2. Our date demonstrates the significant increase in both inflammatory cytokines ((IL)-6 and TNF-ά) in the PPA-induced rodent model of ASD, together with the remarkably similar protective effects of the four different nutritional interventions. IL-10 as an anti-inflammatory cytokine did not significantly demonstrate any changes either with PPA toxicity or any of the nutritional intervention strategies.

## 4. Discussion

The present investigation establishes the efficacy of probiotics and prebiotics as a protective nutritional strategy that may modulate oxidative stress, neuroinflammation, altered gut microbiota, and host immunological responses. Probiotics function in symbiosis with non-digestible oligosaccharides, known as prebiotics, to provide benefits to the brain and central nervous system [30]. Numerous non-randomized studies indicate that some fermented foods may lower a child’s risk of gastrointestinal inflammation [31]. According to Saavedra et al. [32], giving probiotics to hospitalized newborns dramatically decreased the likelihood of GI inflammation.

Because of its high metabolic rate and inadequate antioxidant defense ability, the brain is sensitive to increasing reactive oxygen species ROS. Glutathione peroxidases have also been linked to the protection of a variety of common and complex diseases, including neurological problems [33]. The significant decrease in GPX in the PPA-induced autism rat model could be interpreted as a neurotoxic effect of PPA because GPx protects mammalian cells from oxidative damage by catalyzing the reduction of a range of hydroperoxides (ROOH and H_2_O_2_) to water or the equivalent alcohols utilizing GSH. Numerous studies have been conducted to increase the bioactivity of a variety of dairy products for human health by adding natural ingredients, including herbs [34]. However, no studies on the effects of yogurt enhanced with probiotics as a new dairy product have been conducted. Table 1 and Figure 1 demonstrate the antioxidant effects of yogurt enriched with probiotics or pure *L rhamnosus*. It can be easily observed that both induced GSH and GPX as non-enzymatic and enzymatic antioxidants, respectively. This is in good agreement with the previous work of Choi et al. [35], in which higher brain glutathione levels were linked to higher dairy consumption in older persons. One possible reason for this link is that dairy foods are rich in substrates for glutathione production in the human brain. The highly significant increase in GSH and GPX in artichoke and luteolin-protected rats can be complemented by the work of. Salekzamani et al. [36], which showed that supplementing with artichoke extract increased the levels of the antioxidants catalase, GSH, GPX, and superoxide dismutase (SOD) and decreased the levels of malondialdehyde as a marker of oxidative stress in the liver and plasma of animals with induced disease in comparison to the control group. Indisputable proof of the artichoke’s antioxidant properties in animals was revealed by our current study. 

Animals, plants, and microbes frequently contain the non-protein amino acid GABA. It serves as the central nervous system’s main inhibitory neurotransmitter in animals. GABA is mostly produced by microorganisms including yeast, fungus, and bacteria during fermentation. The majority of lactic acid bacteria (LAB), specifically *Lactococcus lactis, Lactococcus brevis, Lactococcus paracasei,* and *L. plantarum,* create GABA by the enzymatic reaction of glutamate decarboxylase (GAD), a pyridoxal 5′-phosphate (PLP) dependent enzyme, which decarboxylates glutamate. Table 1 shows a striking rise in GABA levels in the yogurt-protected and *L rhamnosus*-protected groups, supporting the idea that both foods have protective effects when used as dietary interventions in ASD mouse models. It is generally known that patients with ASD have significantly disrupted GABAergic signaling. Increased brain GABA is advised to reverse the well-recognized imbalanced glutamate/GABA etiological process in ASD.

Additionally, Table 1 shows the striking GABA drop in PPA-treated mice and the relative GABA restoration in the artichoke-protected group but not in the luteolin-pre-treated group. This may be due to the entire artichoke extract’s high GABA concentration, which was most recently observed by Ingallina et al. [37] The lack of GABA restoration in the luteolin-protected group is consistent with the findings of Raines et al. [38] showing no evidence of luteolin’s ability to reduce anxiety by modulating the GABAA receptor.

In addition to evidence of immunological malfunction and excessive inflammation typically encountered by autistic people, Hughes et al. [39] found immune risk factors for ASD. IL-6 and other innate inflammatory cytokines are frequently elevated in ASD. TNF-ά is the only cytokine that is known to be associated with the severity of ASD symptoms, making it a significant factor in the development of ASD. Patients with ASD may have elevated TNF-ά which can activate inflammatory signaling. Table 2 and Figure 2 demonstrate the significant increases of both inflammatory cytokines IL-6 and TNF-ά in the PPA-induced rodent model of ASD, together with the remarkable protective effects of the four different nutritional interventions. The yoghurt and pure *L. rhamnosus* GG groups, as probiotic-protected groups, and artichoke and luteolin groups, as prebiotic-protected groups, all showed more or less the same percentage decrease of IL-6 and TNF-ά. This would support the idea that ingesting prebiotics can control and restore a balanced microbiota, which produces SCFAs. SCFA, a consequence of a healthier microbiota, has a major impact on intestinal epithelial cells and immune system cells. SCFA contributes to the reduction of inflammation by suppressing pro-inflammatory cytokines. This could reduce the brain inflammation through the gut-brain axis [39]. On the other hand, IL-10 as an anti-inflammatory cytokine was non-significantly demonstrated any changes either with PPA toxicity or any of the nutritional intervention strategies. The observed anti-inflammatory effects of yogurt and the pure lactobacillus strain (Table 2 and Figure 2) are in good agreement with multiple studies in human and animal models. By fermenting milk with Lactobacillus and/or Streptococcus thermophiles and their probiotic strain concentration, yogurt has been shown to have anti-inflammatory benefits [40]. In both animal models and human investigations, there is compelling evidence that probiotics have anti-inflammatory activities [41,42]. According to one study, giving lactic-acid-bacteria-containing yogurt to obese mouse models resulted in significantly lower levels of TNF-α and IL-6 [40].

The previously published work of Ayaz et al. [43] is in good agreement with the documented neuroprotective properties (antioxidant and anti-inflammatory effect) of artichokes and luteolin as a key ingredient of the artichoke, which indicates that artichoke’s high content of phenolic acids and flavonoid glycosides can protect against and slow the onset of Alzheimer’s disease. This is consistent with previously reported research on flavonoids’ capacity to arrest the course of clinical signs of brain disorders by interacting with and altering numerous signaling protein pathways. Artichoke flavonoids have the ability to increase vascular blood flow and suppress neuronal death caused by neurotoxic agents such as PPA.

Furthermore, the reported neuroprotective effects of yogurt and *L. rhamnosus* GG are similar to a previous study by Cheon et al. [44] that found probiotics to be a preventive functional diet with probiotic potential and neuroprotective effects. Multiple lactobacillus species were found to demonstrate a neuroprotective effect against 1-methyl-4-phenyl pyridinium (MPP+)-induced toxicity in SH-SY5Y neuron cells. 

Clinically, probiotic treatment was successful in reducing GI symptoms in kids with ASD, according to studies by Wang et al. and Mohsen et al. [45,46]. The research of Li et al. also revealed [47] that probiotic administration enhanced the relative concentrations of good bacteria (Lactobacillus) in the guts of kids with ASD. Children with ASD exhibit too precise and picky eating habits, which may have an effect on gastrointestinal symptoms and intestinal flora abundance [47]. In assumption, several clinical trials have hypothesized that prebiotics and probiotics might lessen GI symptoms in kids with ASD by raising the relative abundance of helpful bacteria there. Lactobacillus has been shown to ease GI symptoms, which may be connected to the butyric acid in its metabolites [48]. Excitingly, a combination of the flavonoids luteolin and quercetin appeared to be beneficial in reducing ASD symptoms in children while having no major negative effects [49]. Both exhibit potent antioxidant and anti-inflammatory activities, and inhibit the release of inflammatory cytokines from human mast cells [50].

Because gut microbiota can influence brain biochemical and behavioral phenotypes via the microbiome-gut-brain axis, this study provides good evidence that early use of prebiotics and probiotics, particularly luteolin and lactobacillus, as supplements or in food-rich sources could be effective in maintaining and/or restoring the dynamic balance between the microflora and host defense mechanisms in the intestinal mucosa, and thus might help avoid the onset and progression of ASD. Nevertheless, the current study has a flaw that must be addressed in future investigations. The effect of the four dietary therapies on social interaction, an important element of ASD, has not been studied.

## 5. Conclusions

The current study supports the potential neuroprotective effects of probiotic pre-treatment (either yoghurt or pure *Lacticaseibacillus rhamnosus* GG) and prebiotic consumption (artichoke and luteolin) as nutritional intervention strategies to prevent oxidative stress, altered GABA, and neuroinflammation in the form of neurotoxic effects of orally administered PPA, in rodent model of ASD. All therapies demonstrated moderate anti-inflammatory and antioxidant efficacy. As a result, they can be used as a functional dietary component to help avoid ASD.

## 6. Future Perspectives

As a result of the success of using probiotics and prebiotics as supplements or as part of meals rich in foods to lessen induced biochemical autistic features in a rodent model of ASD, we believe that future research will be able to comprehensively assess the physiological effects of these diets. Such information can be used to build interventions that are more informed, less constrictive, free from the negative effects of limiting certain nutrients, and still keep the elements that support the positive behavioral modification of ASD. Moreover, in order to precisely establish the therapeutic effects of the studied nutritional interventions on ASD in children, randomized, double-blind, and placebo-controlled studies adhering to strict trial criteria are required.

## Figures and Tables

**Table 1:** Protective effect of nutrition with yogurt, artichoke, *L. rhamnosus* GG, and luteolin on levels of GPX1, GSH, and GABA in brain homogenates of the PPA-induced rodent model of autism.

Parameters	Groups	Mean ± S.D.	*p* Value
GPX (mU/mg protein)	Control	108.60 ± 11.05	0.001
	PPA	75.19 ± 9.72 ^a^**	
	Yogurt-protected	98.82 ± 12.58 ^b^**	
	Artichoke-protected	99.40 ± 17.30 ^b^**	
	*L rhamnosus* GG-protected	102.82 ± 14.28 ^b^**	
	Luteolin-protected	97.79 ± 17.95 ^b^*	
GSH	Control	15.18 ± 2.27	0.001
	PPA	8.13 ± 1.82 ^a^**	
	Yogurt-protected	11.32 ± 2.14 ^a^**^b^**	
	Artichoke-protected	10.83 ± 1.64 ^a^**^b^*	
	*L rhamnosus* GG-protected	9.24 ± 1.35 ^a^**	
	Luteolin-protected	12.85 ± 1.39 ^a^*^b^**	
GABA (Pg/mg protein)	Control	65.91 ± 10.41	0.004
	PPA	35.80 ± 5.82 ^a^**	
	Yogurt-protected	48.90 ± 12.70 ^a^*	
	Artichoke-protected	46.50 ± 10.55 ^a^**	
	*L rhamnosus* GG-protected	41.52 ± 8.26 ^a^**	
	Luteolin-protected	33.51 ± 14.19 ^a^**	

^a*^ Describes significant difference between the group and the (Control group) below significance level (0.05). ^b*^ Describes significant difference between the group and the (PPA-treated group) below significance level (0.05). ** describes significant difference at significant level (0.01).

**Table 2:** Protective effects of nutrition with yogurt, artichoke, *L. rhamnosus*, and luteolin on levels of TNF-α, IL10, and IL-6 in in brain homogenates of PPA-induced rodent model of autism.

Parameters	Groups	Mean ± S.D.	*p* value
TNF-α (pg/mL)	Control	14.40 ± 3.53	0.001
	PPA	37.53 ± 8.89 ^a^**	
	Yogurt-protected	21.13 ± 2.82 ^b^**	
	Artichoke-protected	21.82 ± 6.41 ^a^*^b^**	
	*L rhamnosus* GG-protected	21.70 ± 4.64 ^a^*^b^**	
	Luteolin-protected	22.60 ± 3.28 ^a^*^b^**	
IL10 (pg/mg protein)	Control	49.74 ± 3.50	0.331
	PPA-pretreated	40.11 ± 6.76	
	Yogurt-protected	46.82 ± 3.76	
	Artichoke-protected	48.42 ± 10.57	
	*L rhamnosus* GG-protected	39.18 ± 1.18	
	Luteolin-protected	41.82 ± 5.00	
IL-6	Control	10.86 ± 0.80	0.001
	PPA-pretreated	47.69 ± 9.33 ^a^**	
	Yogurt-protected	25.91 ± 3.46 ^a^**^b^**	
	Artichoke-protected	26.75 ± 7.86 ^a^**^b^**	
	*L rhamnosus* GG-protected	26.61 ± 5.69 ^a^**^b^**	
	Luteolin-protected	27.72 ± 4.02 ^a^**^b^**	

^a*^ describes significant difference between the group and the (Control group) at significance level (0.05). ^b*^ describes significant difference between the group and the (PPA-treated group) at significance level (0.05). ** describes significant difference at significance level (0.01).

## Data Availability

The datasets of the current study are available from the corresponding author on reasonable request. The data are not publicly available due to privacy.
